# Combined temozolomide, immunotherapy and radiotherapy in a patient with anaplastic oligodendroglioma and multiple extracranial metastases: A rare case report

**DOI:** 10.1097/MD.0000000000045142

**Published:** 2025-10-24

**Authors:** Liang-Ke Tang, Yi Sun, Ming-Hui Zhang, Heng Jiang, Zhi-Ke Li, Guo-Bo Du

**Affiliations:** a Department of Oncology and Hematology, Air Force Hospital of Western Theater Command, Chengdu, China; b Department of Oncology, Affiliated Hospital of North Sichuan Medical College, Nanchong, China.

**Keywords:** 1p/19q chromosome deletions, anaplastic oligodendroglioma, and radiotherapy, immunotherapy, multiple extracranial metastases, temozolomide

## Abstract

**Rationale:**

Extracranial metastases of primary brain tumors are rare, and there is no effective treatment. Here, we report a patient with anaplastic oligodendroglioma (AO, WHO grade III) who effectively delayed survival time after receiving temozolomide (TMZ), immunotherapy, and radiotherapy.

**Patient concerns:**

A 42-year-old man underwent surgery and chemoradiotherapy for AO, 41 months ago.

**Diagnosis:**

The patient developed lower back pain, and Positron emission tomography/computed tomography (PET/CT) did not detect any lesions other than the skeleton. Sacral aspirate smear showed atypic cell nests, and immunohistochemistry and fluorescence in situ hybridization testing supported the diagnosis of WHO grade III AO and IDH mutations.

**Interventions:**

The patient was treated with TMZ, immunotherapy, and local palliative radiotherapy and was stable for 6 months, but the medication was discontinued due to severe myelosuppression.

**Outcomes:**

After drug withdrawal, the disease progressed further, with intracranial recurrence and metastasis to the liver, supraclavicular and axillary lymph nodes. The time from the diagnosis of extracranial metastasis to death was 10 months.

**Lessons:**

This case show that immunotherapy, oral low-dose TMZ and local palliative radiotherapy may be effective ways to prolong the survival of patients with extracranial metastasis and severe bone marrow suppression.

## 1. Introduction

Oligodendrogliomas (ODs) is a malignant tumor of central neuroepithelial origin, accounting for approximately 4.2% of all primary brain tumors.^[1]^ ODs is characterized by recurrent intracranial recurrence, and the five-year survival rate ranges from 32 to 69% worldwide.^[2]^ The main treatments for ODs are surgery, chemotherapy, and radiation. The chromosome 1p/19q codeletion and IDH-1/2 mutant of ODs are thought to be significantly associated with chemotherapy response, longer progression-free survival (PFS), and overall survival (OS).^[3]^ ODs is least likely to develop extracranial metastases compared with other types of gliomas, about approximately 5.25%^[4]^ of extracranial metastases, and the most common sites of metastasis are bone, lymph nodes, and scalp.^[5]^ Among immune checkpoint molecules, programmed death 1 (PD-1)and its ligands PD-L1 have been the most extensively studied. immune checkpoint inhibitors can disrupt the PD-1/PD-L1 interaction, allowing T cells to regain their cytotoxic potential and eliminate tumor cells.^[6]^ The efficacy of immunotherapy is not only associated with PD-L1 expression levels, tumor mutational burden, and tumor-infiltrating lymphocytes, but is also closely linked to patient nutritional status^[7]^ and the timing of immunotherapy intervention.^[8]^ We report a case of anaplastic oligodendroglioma (AO, WHO grade III) containing 1p/19q codeletion and IDH-1 mutant, which spread to the bones and bone marrow 3 years after surgery, and disease progression after chemotherapy, immunotherapy, and radiotherapy, eventually metastasizing to the neck, scalp, and liver.

## 2. Case presentation

**Figure 1.**
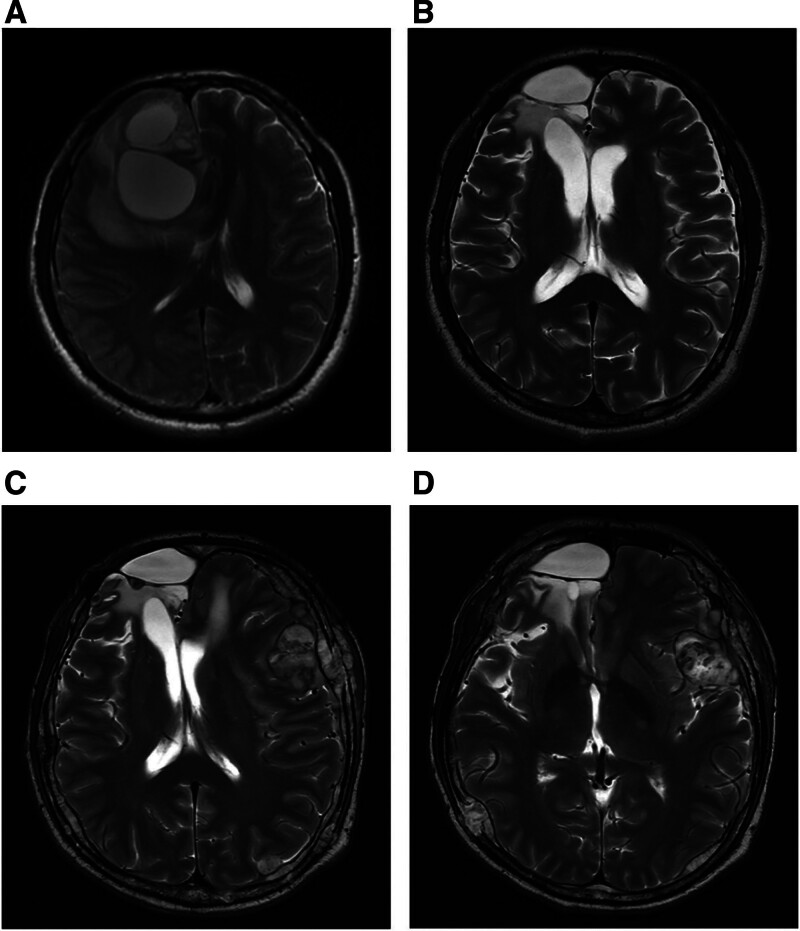
The transverse position T2 images (MRI) of the intracranial primary tumors: (A) Preoperative intracranial lesions on September 2, 2018; (B) No intracranial recurrence was observed on December 13, 2021; (C and D) Metastases were found in meninges, skull, and brain tissues on November 2, 2022. MRI = magnetic resonance imaging.

**Figure 2.**
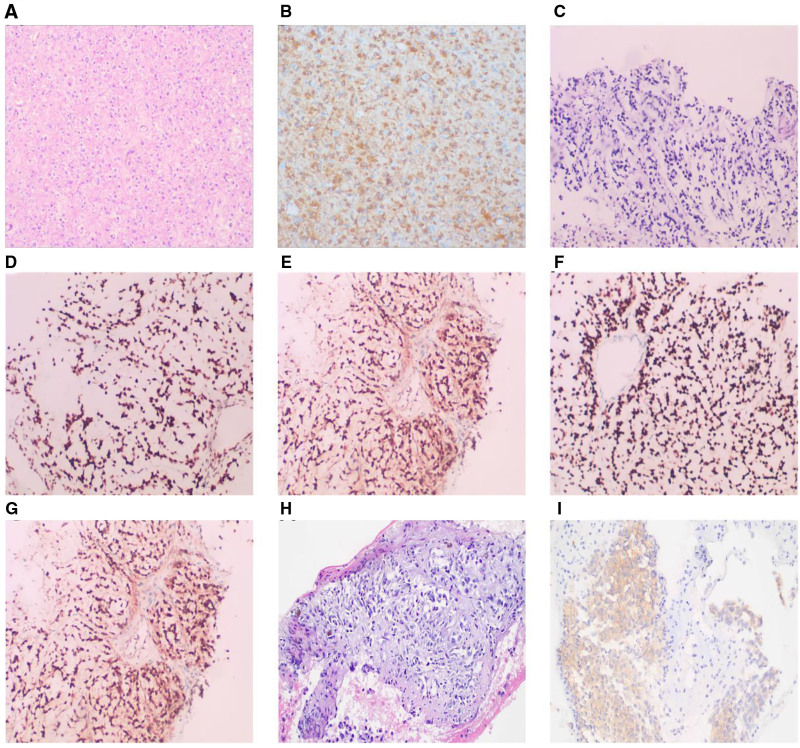
Results of hematoxylin–eosin (HE) staining and immunohistochemistry of the intracranial primary tumors: (A) HE staining, 100×; (B) olig2 (+), 100×. HE staining and immunohistochemistry of the bone marrow aspiration tissue: (C) HE staining, ×100; (D) ATRX (+), ×100; (E) IDH-1 (+), ×100; (F) olig2 (+), ×100; (G) S-100 (+), ×100. HE staining and immunohistochemistry of the supraclavicular lymph nodes aspiration tissue (H) HE staining, ×100; (I) IDH-1, ×100.

A 42-year-old male presented with paroxysmal headache in September 2018, and an magnetic resonance imaging scan revealed a 6.1 cm × 3.5 cm cystic solid mass in the left frontal lobe (Fig. 1A). The mass was removed intactly by craniotomy. Postoperative immunohistochemistry and Fluorescence in situ hybridization (FISH) confirmed the diagnosis of AO and 1p/19q codeletion, (Fig. 2A and B). Gamma Knife radiosurgery and temozolomide (TMZ) chemotherapy were administered. Periodic reviews showed no signs of intracranial tumor recurrence (Fig. 1B).

**Figure 3.**
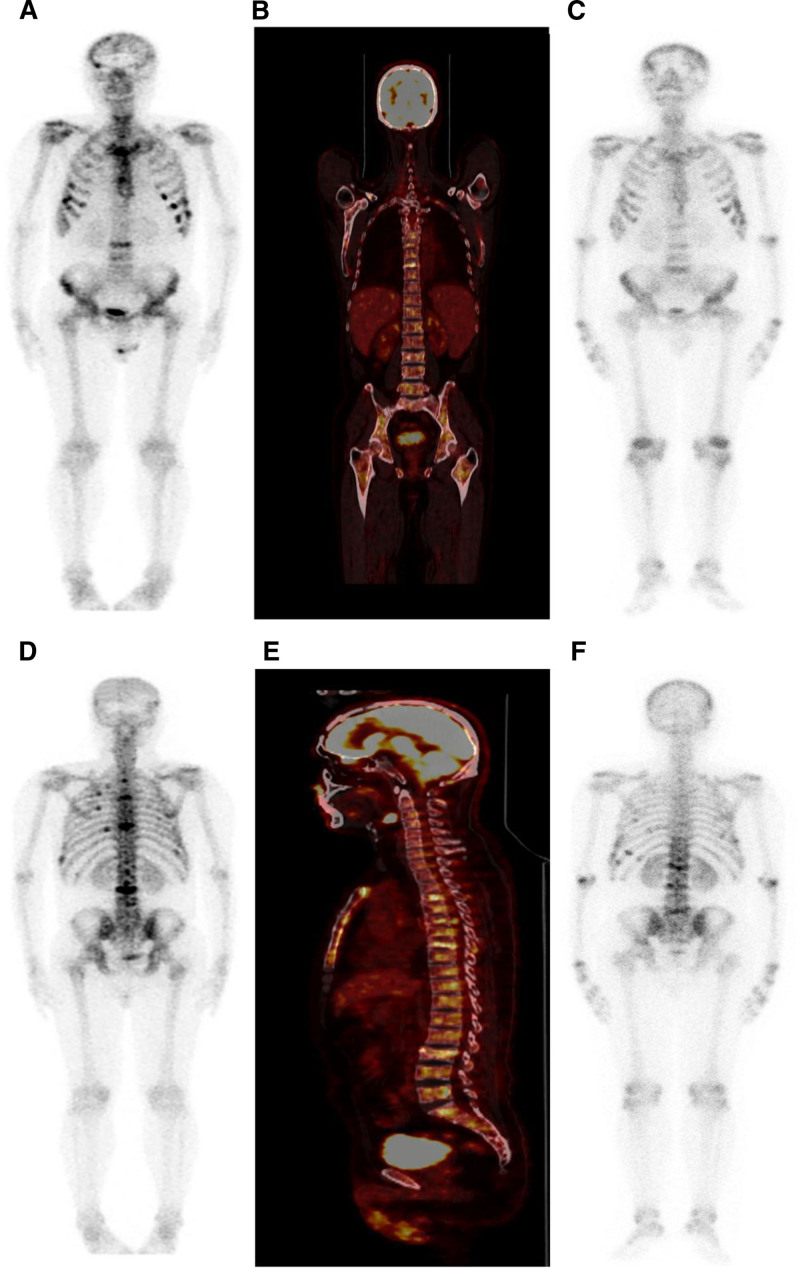
(A and D) Bone scan suggested the involvement of the skull, bilateral scapulae, sternum, and multiple branches of ribs on January 21, 2022. (B and E) PET/CT did not detect any lesions other than those in the bones. (C and F) Bone scans showed fewer lesions in the thoracic spine than before, but a few new lesions in the lumbar spine and ribs on July 27, 2022.

Unfortunately, in January 2022, the patient developed with back pain and a bone scan suggested extensive metastatic bone disease involving of the skull, bilateral scapulae, sternum, and multiple branches of ribs (Fig. 3A and D). Positron emission tomography/computed tomography (PET/CT) did not detect any lesions other than those in the bones (Fig. 3B and E). And a sacral puncture smear revealed nests of heterotypic cells. Immunohistochemistry showed IDH-1 (+), S-100 (+), GFAP (+, weakly positive), ATRX (+), P53 (−), olig2 (+), Ki-67 (+, 20%) and FISH showed 1p/19q codeletion. All results supported the diagnosis of AO, IDH mutant type, WHO grade III (Fig. 2C–G). He was treated with TMZ (250 mg days 1–5) for one cycle on January 28, 2022. But the patient could not tolerate the previous TMZ treatment regimen and developed severe myelosuppression (hemoglobin was 70 g/L, platelet was 20 × 10^9^/L). Then a small oral dose of TMZ (100 mg, days 1–21, repeated every 28 days) and instillation of Toripalimab (peptide inhibitors targeting human programmed death 1 (PD-1) receptor, 240 mg every 21 days) were started with the patient’s consent on March 16, 2022 after the level of platelet counts returned to 50 × 10^9^/L. Simultaneously, the patient received palliative radiotherapy for pelvic, lumbar, and thoracic metastases (P-CTV:30GY/10Fx). After 2 cycles of therapy, severe myelosuppression occurred (platelet was 6 × 10^9^/L, white blood cell was 0.71 × 10^9^/L, and hemoglobin was 44 g/L) On May 16, 2022, TMZ was discontinued while immunotherapy continued.

**Figure 4.**
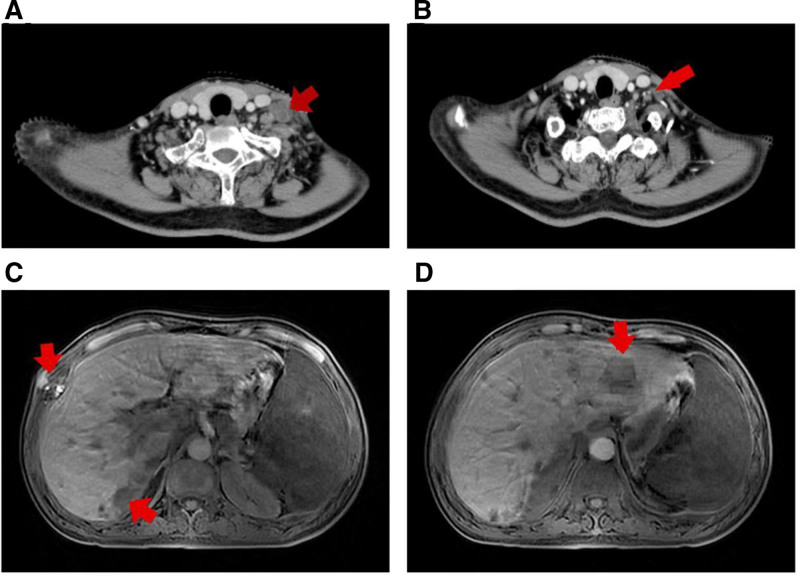
(A) Cervical lymph nodes before radiotherapy on July 27, 2022; (B) Cervical lymph nodes after stage I radiotherapy on August 18, 2022; (C, D) The transverse position T1 images (MRI) of liver and ribs metastases. MRI = magnetic resonance imaging.

In July 2022, bone scans showed fewer lesions in the thoracic spine than before, but a few new lesions in the lumbar spine and ribs (Fig. 3C and F). And, the CT showed enlargement of lymph nodes in the right neck, left supraclavicular, with the largest one measuring 3.0 cm × 1.9 cm (Fig. 4A). Biopsy of supraclavicular lymph nodes was diagnosed as metastatic AO (Fig. 2H and I), and immunotherapy was discontinued. Radiotherapy (the first stage: P-GTVnd:33.6GY/14Fx, P-CTV:26.6Gy/14Fx, the second stage: P-GTVnd:32.5GY/13Fx, P-CTV:26Gy/13Fx) was recommended for positive lymph nodes and lymph node area in the neck and the patient’s neck mass was significantly smaller after the radiotherapy (Fig. 4B). Oral TMZ was again initiated (100 mg) on August 25, 2022, but the patient’s platelets rapidly declined from 50 × 10^9^/L to 32 × 10^9^/L after 4 days and then TMZ was discontinued.

In November 2022, magnetic resonance imaging showed multiple patchy and nodular abnormal signal shadows in the skull and the meninges were thickened with nodular and mass-like changes, and the left anterior horn of the lateral ventricle was compressed (Fig. 1C and D). Multiple nodular abnormal signal shadows were seen in the liver, with a size of about 2.0 cm and poorly defined borders (Fig. 4C and D). Best supportive care was administered until the patient’s death on November 20, 2022. The patient was diagnosed with bone metastases and was treated with TMZ, immunotherapy, and radiotherapy to stabilize the disease for 6 months. Then the disease progressed rapidly and died 4 months later.

## 3. Discussion

Extracranial metastases from primary brain tumors are rare, accounting for only 0.96%.^[9]^ Glioblastoma and medulloblastomas are the most common brain tumors known to metastasize to distant sites, while ODs occur less frequently. Both high-grade and low-grade ODs have the potential to metastasize to other sites, the most common sites being the bone and bone marrow (97%), followed by lymph nodes, lungs, and pleura.^[10]^ Primary tumors in the brain may spread through local invasion, seeding through the cerebrospinal fluid route, or remote dissemination through lymphatic and vascular channels. In this patient, the metastasis occurred potentially through the hematologic and lymphatic systems due to the sequential metastases in the liver and lymph nodes. Surgical treatment or extracranial shunts may disrupt the dura mater and increase the risk of hematologic and lymphatic metastases, extracranial metastases without surgery are rare.^[11]^ This may be an illusion caused by the small number of unoperated patients.^[12,13]^ Some scholars believe that advances in treatment had prolonged the survival of patients and led to an increased occurrence of extracranial metastases. However, the epidemiological studies didn’t support it.^[14]^

The diagnosis of oligodendroglioma primarily relies on pathological biopsy. it is genetically characterized by the presence of an IDH-1 or IDH-2 mutation combined with co-deletion of 1p/19q codeletion. Histologically, these tumors typically exhibit sheets of uniform round nuclei surrounded by clear cytoplasm, resulting in the classic “fried egg” appearance. AO are associated with a poorer prognosis due to the presence of anaplastic features, including nuclear atypia, necrosis, microvascular proliferation, high cellular density, and increased mitotic activity. Heterozygous chromosome 1p/19q codeletion was associated with significantly longer PFS in patients with AO, with a median PFS of 49.8 months and only 6.2 months in PFS without 1p/19q.^[15]^ Longer survival may be associated with mutations in the far upstream (FUSE) element-binding protein 1 (FUBP1), located on chromosome 1p. FUBP1 is a DNA-binding and transcriptional regulatory protein, and mutations in FUBP1 are observed in approximately 15 to 30% of tumors with 1p/19q codeletion.^[16]^ FUBP1 can promote MYC expression by binding to the FUSE region of the proto-oncogene MYC.^[17,18]^ The extended survival associated with 1p/19q codeletion may be closely related to extracranial metastasis.^[19,20]^ In this case, the patient’s PFS was 41 months and both intracranial primary lesions and extracranial metastases had 1p/19q codeletion. There is still the need for further studies to determine whether longer survival and the 1p/19q codeletion are associated with extracranial metastases.

There is no standard treatment option for extracranial metastatic oligodendroglioma. Procarbazine, lomustine, and vincristine (PCV) or TMZ therapy remains effective.^[21]^ Patients with bone marrow metastases and severe myelosuppression have short survival, which may be due to the lack of further treatment option.^[5,22]^ However, A report shows that extracranial metastatic oligodendroglioma with BRAF-V600e gene mutation can be treated with targeted therapy, effectively prolonging the survival of patients with no significant adverse effects.^[23]^ In the present case, no gene target was found, and the patient did not receive any drugs that caused myelosuppression before the metastasis was diagnosed. A small dose of TMZ was given in combination with immunotherapy, which was effective in controlling the disease. Extracranial metastases are not limited by the blood-brain barrier, which allows immunotherapy to enhance the diversity of immune cell populations. Future directions in oligodendroglioma immunotherapy will likely focus on overcoming immunosuppressive mechanisms within the tumor microenvironment (TME), as well as developing novel immunotherapies that target tumor-specific mutant molecules with anti-tumor potential. A promising strategy involves combining IDH-1 neoantigen vaccines with PD-1/PD-L1 checkpoint inhibitors to counteract TME-mediated immunosuppression.^[24]^

However, the patient could no longer tolerate the toxic side effects of TMZ and thus discontinued the treatment. The disease then progressed rapidly, metastasizing to the scalp and liver, eventually leading to death. The limitation of this study is that no autoantibody testing and bone marrow aspirate/biopsy are performed when patients develop bone marrow suppression to further determine whether platelet and hemoglobin reductions are related to immunotherapy. In addition, comprehensive genomic sequencing was not conducted for this patient, thus preventing further investigation into whether the extracranial metastases were related to specific genetic alterations.

## 4. Conclusion

In conclusion, this case is a rare extracranial metastasis of AO that successively invaded bone, bone marrow, liver, and scalp. The present case suggests that TMZ therapy is effective in the treatment of extracranial oligodendroglioma and that low-dose TMZ, radiotherapy and immunotherapy are effective in delaying disease progression in cases of severe thrombocytopenia due to bone marrow invasion.

## Author contributions

**Conceptualization:** Guo-Bo Du.

**Data curation:** Heng Jiang.

**Supervision:** Yi Sun.

**Writing – original draft:** Liang-Ke Tang.

**Writing – review & editing:** Ming-Hui Zhang, Zhi-Ke Li.

## References

[1] Leonardi MA Lumenta CB. Oligodendrogliomas in the CT/MR-era. Acta Neurochir (Wien). 2001;143:1195–203. 11810382 10.1007/s007010100014

[2] Girardi F Matz M Stiller C; CONCORD Working Group. Global survival trends for brain tumors, by histology: analysis of individual records for 556,237 adults diagnosed in 59 countries during 2000–2014 (CONCORD-3). Neuro Oncol. 2023;25:580–92. 36355361 10.1093/neuonc/noac217 PMC10013649

[3] Brandes AA Tosoni A Cavallo G; GICNO. Correlations between O6-methylguanine DNA methyltransferase promoter methylation status, 1p and 19q deletions, and response to temozolomide in anaplastic and recurrent oligodendroglioma: a prospective GICNO study. J Clin Oncol. 2006;24:4746–53. 16954518 10.1200/JCO.2006.06.3891

[4] Liwnicz BH Rubinstein LJ. The pathways of extraneural spread in metastasizing gliomas: a report of three cases and critical review of the literature. Hum Pathol. 1979;10:453–67. 381159 10.1016/s0046-8177(79)80051-9

[5] Singh VK Singh S Bhupalam L. Anaplastic oligodendroglioma metastasizing to the bone marrow: a unique case report and literature review. Int J Neurosci. 2019;129:722–8. 30526175 10.1080/00207454.2018.1557165

[6] Ribas A Wolchok JD. Cancer immunotherapy using checkpoint blockade. Science. 2018;359:1350–5. 29567705 10.1126/science.aar4060 PMC7391259

[7] Vitale E Maistrello L Rizzo A. Nutritional conditions and PFS and OS in cancer immunotherapy: the MOUSEION-010 meta-analysis. Immunotherapy. 2025;17:269–81. 40134096 10.1080/1750743X.2025.2483656 PMC12013447

[8] Rizzo A Monteiro FSM Mollica V. Impact of time-of-day administration of immunotherapy on survival in metastatic renal cell carcinoma: the MOUSEION-09 meta-analysis. Clin Exp Metastasis. 2025;42:3. 10.1007/s10585-024-10322-1 39680251

[9] Schweitzer T Vince GH Herbold C Roosen K Tonn JC. Extraneural metastases of primary brain tumors. J Neurooncol. 2001;53:107–14. 11716064 10.1023/a:1012245115209

[10] Dawson TP. Pancytopaenia from a disseminated anaplastic oligodendroglioma. Neuropathol Appl Neurobiol. 1997;23:516–20. 9460719 10.1111/j.1365-2990.1997.tb01330.x

[11] Hoffman HJ Duffner PK. Extraneural metastases of central nervous system tumors. Cancer. 1985;56:1778–82. 4027909 10.1002/1097-0142(19851001)56:7+<1778::aid-cncr2820561309>3.0.co;2-i

[12] McLemore MS Bruner JM Curry JL Prieto VG Torres-Cabala CA. Anaplastic oligodendroglioma involving the subcutaneous tissue of the scalp: report of an exceptional case and review of the literature. Am J Dermatopathol. 2012;34:214–9. 22157246 10.1097/DAD.0b013e318230655c

[13] Ozişik PA Işikay I Oruçkaptan H Söylemezoğlu F Ozcan OE. Unusual massive spinal metastasis of an intracranial oligodendroglioma. Turk Neurosurg. 2008;18:276–80. 18814118

[14] Sharma A Agarwal A Sharma MC Anand M Agarwal S Raina V. Bone marrow metastasis in anaplastic oligodendroglioma. Int J Clin Pract. 2003;57:351–2. 12800473

[15] Bauman GS Ino Y Ueki K. Allelic loss of chromosome 1p and radiotherapy plus chemotherapy in patients with oligodendrogliomas. Int J Radiat Oncol Biol Phys. 2000;48:825–30. 11020580 10.1016/s0360-3016(00)00703-3

[16] Wesseling P van den Bent M Perry A. Oligodendroglioma: pathology, molecular mechanisms and markers. Acta Neuropathol. 2015;129:809–27. 25943885 10.1007/s00401-015-1424-1 PMC4436696

[17] Zhang F Xiong Q Wang M Cao X Zhou C. FUBP1 in human cancer: Characteristics, functions, and potential applications. Transl Oncol. 2024;48:102066. 39067088 10.1016/j.tranon.2024.102066 PMC11338137

[18] Zhao Y Yu Y Chen W Zhang X Lv J Zhao H. Oligodendroglioma: advances in molecular mechanisms and immunotherapeutic strategies. Biomedicines. 2025;13:1133. 40426960 10.3390/biomedicines13051133 PMC12108979

[19] Wang M Murphy KM Kulesza P. Molecular diagnosis of metastasizing oligodendroglioma: a case report. J Mol Diagn. 2004;6:52–7. 14736827 10.1016/S1525-1578(10)60491-6 PMC1867467

[20] Li G Zhang Z Zhang J. Occipital anaplastic oligodendroglioma with multiple organ metastases after a short clinical course: a case report and literature review. Diagn Pathol. 2014;9:17. 24447608 10.1186/1746-1596-9-17 PMC3943380

[21] Mazza E Belli C Terreni M. Breast metastases from oligodendroglioma: an unusual extraneural spread in two young women and a review of the literature. Crit Rev Oncol Hematol. 2013;88:564–72. 23953683 10.1016/j.critrevonc.2013.07.010

[22] Demeulenaere M Duerinck J DU Four S Fostier K Michotte A Neyns B. Bone marrow metastases from a 1p/19q co-deleted oligodendroglioma – a case report. Anticancer Res. 2016;36:4145–9. 27466523

[23] Shi L Zou Z Ding Q. Successful treatment of a BRAF V600E-mutant extracranial metastatic anaplastic oligoastrocytoma with vemurafenib and everolimus. Cancer Biol Ther. 2019;20:431–4. 30462564 10.1080/15384047.2018.1529115 PMC6422448

[24] Bianconi A Palmieri G Aruta G. Updates in glioblastoma immunotherapy: an overview of the current clinical and translational scenario. Biomedicines. 2023;11:1520. 37371615 10.3390/biomedicines11061520 PMC10295612

